# The relationship between physical exercise and depressive symptoms in college students: the mediating role of rumination

**DOI:** 10.3389/fpsyt.2024.1501996

**Published:** 2024-12-05

**Authors:** Bei Zhu, Qing Liu, Shuqi Jia, Xing Wang, Qin Man

**Affiliations:** ^1^ China College of teacher education, East China Normal University, Shanghai, China; ^2^ School of Physical Education, Shanghai University of Sport, Shanghai, China; ^3^ School of Sports and Health, Shanghai Linxin Accounting and Finance University, Shanghai, China

**Keywords:** depressive symptoms, physical exercise, rumination, mediating effect, relationship

## Abstract

**Objective:**

This study aims to explore the correlation between physical exercise, rumination, and depressive symptoms in college students, as well as to investigate the potential pathways through which physical exercise may impact depressive symptoms. This exploration offers valuable insights for the development of clinical exercise interventions.

**Methods:**

A cross-sectional study design was employed, with 2,902 participants recruited via convenience sampling. Structural equation modeling was utilized to explore the relationship between physical exercise and depressive symptoms in college students.

**Results:**

1) Statistically significant differences were observed between depressed and non-depressed college students in terms of rumination, symptom rumination, reflective pondering, and compulsive meditation (all P < 0.05); 2) Physical exercise was found to negatively predict symptom rumination (B=-0.083, P<0.001), compulsive thinking (B=0.034, P>0.05), reflective pondering (B=-0.038, P<0.01), and BDI-II scores (B=-0.103, P<0.001). Symptom rumination positively predicted BDI-II scores (B=0.648, P<0.001), while compulsive thinking and reflective pondering were found to predict BDI-II scores positively (B=0.028, P>0.05) and negatively (B=-0.041, P>0.05), respectively. 3) Physical exercise exerted a direct effect of 59.09% on BDI-II scores (B: -0.065, 95% CI -0.104, -0.028), indicating that higher levels of physical exercise were associated with lower BDI-II scores. The coefficients for duration, intensity, and frequency were statistically significant (all P < 0.05), with intensity and frequency exhibiting higher path coefficients. Rumination, as a latent variable, mediated 40.91% of the indirect effect (B: -0.045, 95% CI -0.077, -0.015), with symptom rumination emerging as a statistically significant pathway (P < 0.05).

**Conclusion:**

Rumination may mediate the relationship between physical exercise and depressive symptoms, with varying mediating effects across different dimensions of rumination. The intensity and duration of physical exercise exert a stronger influence.

## 1 Introduction

Depression is a common mood disorder among college students, often characterized by prolonged periods of persistent sadness, which may even lead to suicidal thoughts (1). College students are a unique group in society, at a specific developmental milestone that connects the critical transition from adolescence to adulthood (2). They face numerous challenges, such as sustained academic pressure (3)、changes in living arrangements and lifestyle (4)、as well as financial or employment pressures (5). In recent years, the detection rate of depression among college students has steadily increased, with a meta-analysis revealing an overall prevalence rate of depression among college students at 28.4% (6). Depression symptoms in college students not only affect their social functioning and academic performance but may also have an impact on their physical health (7, 8).

Rumination is a coping mechanism that focuses on negative emotions, characterized by self-reflection and repetitive, passive attention to negative feelings (9). It is considered a more harmful emotion regulation strategy compared to others, such as acceptance, problem-solving, reappraisal, or suppression. Failure to effectively manage emotions over time can lead to diagnosable depression or anxiety (10). Research shows that rumination affects other mental disorders, such as post-traumatic stress disorder, eating disorders, and sleep disturbances (11, 12). Rumination can contribute to depression in various ways. A study on adolescents with internalizing disorders found that rumination is a predictor of depression risk. Persistent focus on negative events can lead to behavioral and emotional abnormalities, resulting in depression (13). Previous studies have shown that long-term exercise programs can reduce rumination (14). For individuals who do not exercise regularly, acute exercise can boost positive mood effects, but regular exercise may be needed to reduce rumination and negative memory biases. Over time, this may alleviate depressive symptoms (15). Therefore, whether rumination is a mechanism of change in exercise-based treatments for depression requires further investigation.

Physical exercise has been used as an effective method for alleviating depressive symptoms (16). Exercise improves mood by releasing dopamine and endorphins (17), reduces cortisol to alleviate stress and depressive symptoms (18), and promotes metabolism and energy expenditure (19), thus having an antidepressant effect. However, not all studies have confirmed the positive effects of exercise on improving depressive symptoms in children and adolescents. Previous meta-analyses have shown that most studies support the positive impact of exercise on depressive symptoms in college students (20, 21). Additionally, the mediating role of rumination in the relationship between exercise and depressive symptoms is still unclear.

Through a comprehensive review of existing literature, we have identified several critical issues that warrant further investigation: Is there a significant difference in rumination and physical exercise between college students experiencing depressive symptoms and their non-depressed counterparts? Is there a correlation among physical exercise, rumination, and depressive symptoms in college students? If a correlation is established, can rumination serve as a mediator in the relationship between physical exercise and depressive symptoms? Among the three subdimensions—symptom rumination, compulsive thinking, and reflective pondering—which of these dimensions can mediate this relationship? Consequently, this study employs a cross-sectional design to analyze the correlations among physical exercise, rumination, and depressive symptoms in college students, while exploring the potential pathways through which physical exercise influences depressive symptoms The findings of this study will provide valuable insights for the development of clinical exercise programs.

## 2 Participants and methods

### 2.1 Study participants

A convenience sampling method was used to select non-sports students enrolled at a university in Songjiang District, Shanghai, in April 2023. Inclusion criteria: ① Aged 18–25, enrolled university students; ② normal or corrected binocular vision, with no color blindness or color weakness, right-handed, and native Mandarin speakers; ③ voluntary participation in this experimental study. Exclusion criteria: ① dependence on alcohol or drugs, presence of chronic neurological disorders, or history of traumatic brain injury; ② recent use of antidepressant medications such as estazolam, diazepam, or phenobarbital; and ③ physical discomfort or recent sports injuries preventing participation in physical exercise.

### 2.2 Sample size estimation

Based on the Monte Carlo power analysis method for mediation effects, sample size estimation was conducted using the pwrSEM software (available at yilinandrewang.shinyapps.io/pwrSEM/). This process involved defining the model, visualization, setting parameter values, and estimating statistical power. Effect size was determined according to previous literature (22, 23), with the significance level (α) set at 0.05 and 5,000 simulations performed. With a sample size of 2,000, statistical power for detecting mediation effects exceeded 0.8. Considering a 10% attrition rate, an initial sample size of 2,200 was targeted. However, 2,902 participants were ultimately included in the study.

### 2.3 Testing procedure

The tests were conducted between 13:30 and 16:30.

Questionnaires were distributed to participants. Before filling them out, the principal investigator read the instructions, explained the items, clarified that the data collected would only be used for scientific research, and emphasized truthful, independent, and voluntary responses. During the process, participants were reminded to answer carefully as required. Upon completion, invalid questionnaires—those with trap questions answered incorrectly, consecutively skipped items, or regularity in responses—were excluded by the investigators, as well as questionnaires with total physical activity levels beyond three standard deviations. All participants were informed about the testing procedure and the informed consent form. The study received ethical approval, and informed consent was obtained in accordance with the Helsinki Declaration. The study was reviewed and registered by the Ethics Committee of Shanghai University of Sport, under registration number 102772023RT075.

### 2.4 Testing instruments

#### 2.4.1 Physical Activity Rank Scale-3 (PARS-3)

The measurement of physical exercise was conducted using the Physical Activity Rank Scale revised by Liang Deqing (1994). It examines the amount of exercise from three aspects: intensity, duration, and frequency of participation in physical exercise. The total score = exercise intensity × (exercise duration - 1) × exercise frequency, with higher scores indicating greater exercise volume. In this study, the Cronbach’s α for this scale is 0.82 (24).

#### 2.4.2 Ruminative Responses Scale (Nolen-Hoeksema Ruminative Responses Scale, RRS)

The measurement of rumination was conducted using the Ruminative Responses Scale developed by Nolen-Hoeksema (1991). The scale includes three dimensions: symptom rumination, reflective pondering, and compulsive meditation, with a total of 22 items. A 4-point scale is used, with higher scores indicating a higher tendency to ruminate. In this study, the Cronbach’s α for this scale is 0.961 (25).

#### 2.4.3 Beck DePression Inventory, BDI-II

Used to assess the severity of depressive symptoms over the past two weeks. It contains 21 items, rated on a 4-point scale, with total scores ranging from 0 to 63. The scale can be used for clinical diagnosis, with higher scores indicating more severe depression. This study uses a BDI score of >13 as the cutoff for distinguishing depression. In this study, the Cronbach’s α for this scale is 0.913 (26).

### 2.5 Mathematical statistics

Data analysis was conducted using SPSS 29.0 and Amos 24.0. Normally or approximately normally distributed quantitative data were described as M ± SD, and group comparisons were performed using one-way ANOVA. Quantitative data with significant skewness were described as medians (lower quartile, upper quartile), and group comparisons were conducted using the Kruskal-Wallis test. Linear regression analysis or Pearson correlation analysis was used to explore the relationships between physical exercise, rumination, and depressive symptoms. All statistical inferences were performed using two-tailed tests, with a significance level (α) of 0.05. The Harman single-factor test was used to check for common method bias, and a structural equation model was constructed to examine the role of rumination factors in the relationship between college students’ physical exercise levels and depressive symptoms. All variables were standardized before modeling. The model evaluation indices used were the root mean square error of approximation (RMSEA), goodness of fit index (GFI), normed fit index (NFI), and comparative fit index (CFI). Path analysis parameter estimation was performed using the non-parametric percentile Bootstrap method (which does not impose strict requirements on variable distributions), with 5000 resamples. A mediated effect was considered statistically significant if the bias-corrected 95% confidence interval for the product of the mediated path did not include 0.

## 3 Results

### 3.1 Differences in basic information and physical activity levels of college students with varying levels of depressive symptoms

A total of 2902 participants were included, with an average age of 19.40 ± 1.62 years, an average height of 169.24 ± 8.16 cm, and an average weight of 62.16 ± 13.32 kg. Among them, 46.14% were male, 46.62% were only children, 59.44% had urban household registration. The total physical exercise score was 15.34 ± 16.97. The overall depression score was 47.401 ± 10.562, with 533 participants (18.27%) showing signs of depression. Among them, 270 participants (9.30%) had mild depression, 205 participants (7.06%) had moderate depression, and 58 participants (1.99%) had severe depression. College students with depressive symptoms (533 participants) and those with non-depressed (2369 participants) showed significant differences in physical exercise (P < 0.05), and there were significant differences in being only children (P < 0.05). These findings suggest that special attention should be paid to college students’ only child status, and the impact of physical exercise on depressive symptoms. No significant differences were found for other demographic variables (all P > 0.05). Specific differences in demographic variables are shown in **Table 1**.

**Table 1:** Demographic variable differences among college students with different levels of depressive symptoms.

Variable	Total (n=2902)	Depressed (n=533)	Non-Depressed (n=2369)	Difference Test
Age (years)	19.40 ± 1.62	19.60 ± 2.18	19.35 ± 1.46	t=-3.203, *P*=0.078
Height (cm)	169.23 ± 8.16	168.71 ± 8.15	169.36 ± 8.17	t=1.655, *P*=0.098
Weight (kg)	62.15 ± 13.32	61.99 ± 13.91	62.19 ± 13.19	t=0.318, *P*=0.751
BMI(kg·m^-2^)	21.59 ± 3.79	21.58 ± 3.74	21.67 ± 4.02	t=-0.490, *P*=0.624
Gender (male/%)	46.14	43.34	46.77	χ2 = 2.061, P=0.082
Without siblings (Yes/%)	46.62	42.21	47.62	χ2 = 5.100, P=0.024
Household Location (Urban/%)	59.44	55.91	60.24	χ^2^ = 3.378, *P*=0.071

### 3.2 Differences in physical exercise and rumination between depressed and non-depressed college students

Based on BDI-II scores, the participants were divided into depressed and non-depressed college students. The study found statistically significant differences in rumination, symptom rumination, reflective pondering, and compulsive meditation between depressed and normal college students (all P < 0.05). This indicates that college students with depressive symptoms had higher rumination scores and engaged in less physical exercise compared to their non-depressed peers. See **Table 2** for details.

**Table 2:** Differences in physical exercise and rumination between depressed and non-depressed college students.

Variable	Overall (n=2902)	Depressed (n=533)	Non-Depressed (n=2369)	Difference Test
Physical Exercise	15.34 ± 16.97	13.03 ± 16.38	15.86 ± 17.06	t=-3.489, *P*=0.010
Symptom Rumination	40.14 ± 12.99	53.79 ± 12.95	37.07 ± 10.84	t=30.97, *P*<0.001
Reflective Pondering	20.82 ± 7.17	28.78 ± 7.14	19.03 ± 5.83	t=33.34, *P*<0.001
Compulsive Meditation	9.39 ± 3.30	12.08 ± 3.28	8.78 ± 2.99	t=22.59, *P*=0.004
Depressive Symptoms	9.92 ± 3.37	12.92 ± 3.46	9.25 ± 2.96	t=25.05, *P*<0.001

### 3.3 Analysis of differences in rumination and depressive symptoms among college students with different levels of physical exercise

One-way ANOVA revealed significant differences in rumination, its sub-factors, and depressive symptoms among college students with different levels of physical exercise. Students with low levels of physical exercise had higher rumination scores and its sub-factors compared to those with high levels of exercise. Additionally, students with low levels of physical exercise had higher BDI-II scores compared to those with moderate and high levels of exercise. This indicates that the higher the level of physical exercise, the lower the rumination and depressive symptoms among college students. See **Table 3** for details.

**Table 3:** Analysis of differences in rumination and depressive symptoms among college students with different levels of physical exercise.

Variable	Physical Exercise Level			*F*	*Post-hoc* Multiple Comparisons
	Low	Medium	High		
	n=2124	n=522	n=256		
Rumination	40.59 ± 12.82	38.42 ± 12.71	39.90 ± 14.58	5.914**	1>2
Symptom Rumination	21.12 ± 7.11	19.71 ± 7.93	20.53 ± 7.93	8.357***	1>2
Reflective Pondering	9.46 ± 3.24	9.09 ± 3.35	9.39 ± 3.64	2.649	1>2
Compulsive Meditation	9.99 ± 3.32	9.60 ± 3.34	9.96 ± 3.81	2.818	1>2
Depressive Symptoms	7.57 ± 8.20	5.92 ± 6.63	6.23 ± 7.76	11.008***	1>2, 1>3

*, **, *** represent P<0.05, P<0.01, and P<0.001, respectively; in the multiple comparisons, 1 represents low physical exercise levels, 2 represents moderate physical exercise levels, and 3 represents high physical exercise levels.

### 3.4 Relationship between physical exercise, rumination, and depressive symptoms

**Figure 1:**
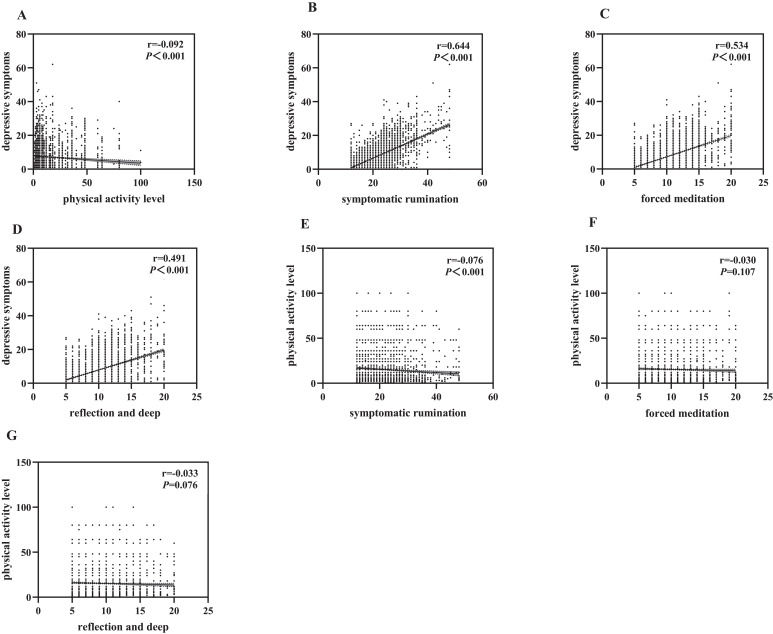
Correlations between variables. **(A)** Depressive symptoms are significantly negatively correlated with physical exercise. **(B)** Depressive symptoms are significantly positively correlated with symptom rumination. **(C)** Depressive symptoms are significantly positively correlated with compulsive meditation. **(D)** Depressive symptoms are significantly positively correlated with reflective pondering. **(E)** Physical exercise is significantly negatively correlated with symptom rumination. **(F)** Physical exercise is positively correlated with compulsive thinking, but not significantly. **(G)** Physical exercise is positively correlated with reflective pondering, but not significantly.

Depressive symptoms were significantly negatively correlated with physical exercise (r=-0.092, P<0.001) and significantly positively correlated with symptom rumination (r=0.644, P<0.001), compulsive meditation (r=0.534, P<0.001), and reflective pondering (r=0.491, P<0.001). Physical exercise was significantly negatively correlated with symptom rumination (r=-0.076, P<0.001) and negatively correlated with compulsive thinking (r=-0.030, P=0.107) and reflective pondering (r=-0.033, P=0.076), though these were not statistically significant. See **Figure 1**.

### 3.5 Common method variance bias test

The Harman single-factor test was used to measure the degree of common method variance (27). The results showed that there were six factors with eigenvalues greater than 1, and the first factor explained 32.50% of the variance, which is below the critical threshold of 40%. Therefore, there is no common method bias issue, and the results are acceptable.

### 3.6 Construction and validation of the structural relationship model

**Figure 2:**
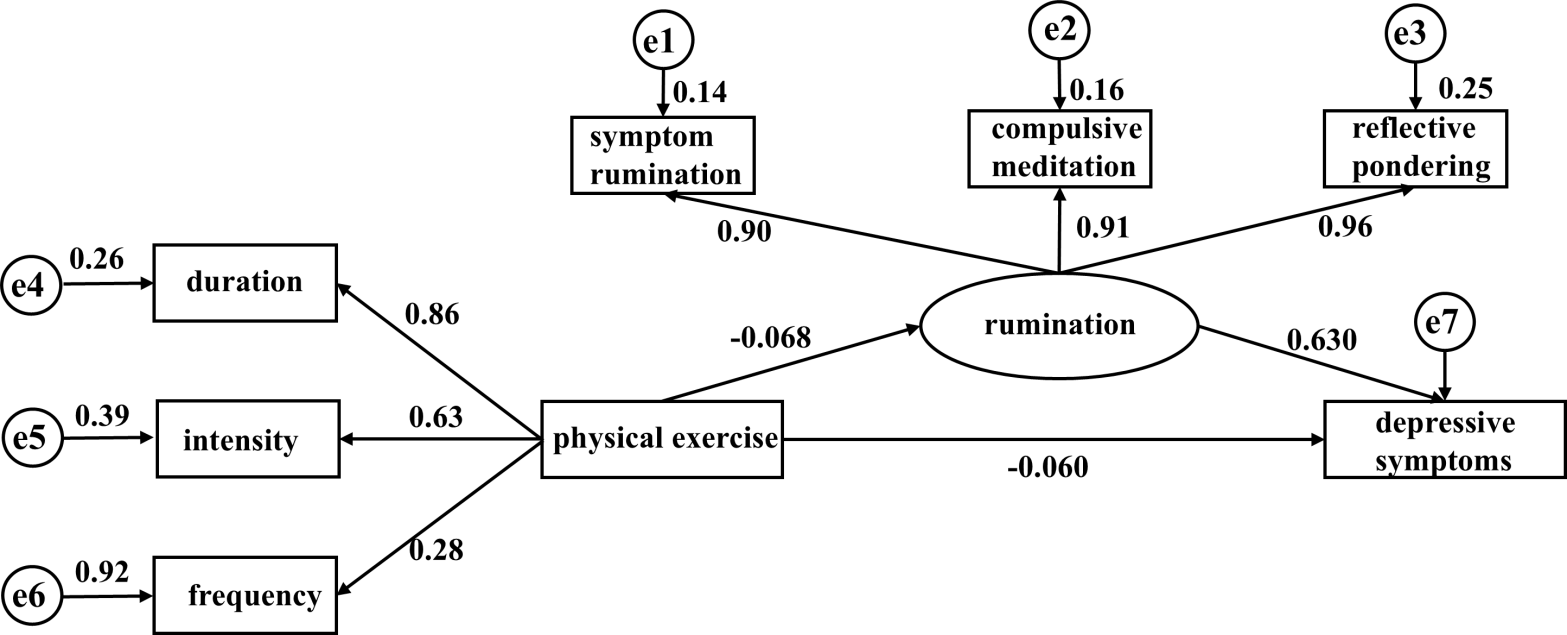
Structural relationship model of physical exercise, symptom rumination, compulsive thinking, reflective pondering, and depressive symptoms (N=2902). Path values are standardized coefficients.

Based on the relationship between physical exercise, rumination, and depressive symptoms, the physical exercise score was used as the independent variable and the BDI-II score as the dependent variable. Model 4 in PROCESS was used to analyze the mediating effects of symptom rumination, compulsive thinking, and reflective pondering. The results showed that physical exercise negatively predicted symptom rumination (B=-0.083, P<0.001), compulsive thinking (B=0.034, P>0.05), reflective pondering (B=-0.038, P<0.01), and BDI-II scores (B=-0.103, P<0.001). Symptom rumination positively predicted BDI-II scores (B=0.648, P<0.001), compulsive thinking positively predicted BDI-II scores (B=0.028, P>0.05), and reflective pondering negatively predicted BDI-II scores (B=-0.041, P>0.05). See **Figure 2** for details.

Based on the relationship between physical exercise, rumination, and depressive symptoms, a structural equation model was established with physical exercise as the independent variable, BDI-II score as the dependent variable, and the factors of rumination as mediators. The model showed good fit (χ2/df=2.689, RMSEA=0.024, GFI=0.993, NFI=0.997, CFI=0.998). Path analysis is shown in the figure, and the mediation test results are shown in the table. Physical exercise had a direct effect of 59.09% on BDI-II scores (B: -0.065, 95% CI -0.104, -0.028), indicating that the higher the level of physical exercise, the lower the BDI-II scores. The coefficients of the duration, intensity, and frequency paths were all statistically significant (all P < 0.05), with intensity and frequency having higher path coefficients. Rumination, as a latent variable, mediated 40.91% of the mediation effect (B: -0.045, 95% CI -0.077, -0.015), with the symptom rumination path having statistical significance (P < 0.05). This result suggests that rumination may mediate the relationship between physical exercise and depressive symptoms, acting as a partial mediator, with intensity and frequency playing stronger roles. See **Table 4** for details.

**Table 4:** Bootstrap analysis of the significance test for mediation effects (N=2902).

Effect Type	*B*	Bias-Corrected 95% CI	*P-value*	*SE*	Proportion of Effect (%)
Total Effect	-0.110	(-0.161, -0.063)	<0.001	0.025	100
Direct Effect	-0.065	(-0.104, -0.028)	0.005	0.016	59.09
Indirect Effect	-0.045	(-0.077, -0.015)	0.005	0.124	40.91

*B*, Mediation Effect Coefficient; CI, Confidence Interval; SE, Standard Error.

## 4 Discussion

The findings of this study reveal a significant association between depressive symptoms, rumination, and physical exercise among college students. Higher levels of physical exercise are associated with reduced rumination and lower depressive symptomatology. This finding further substantiates the positive impact of physical exercise in mitigating rumination and alleviating depressive symptoms. Rumination and depressive symptoms demonstrated a positive correlation, with symptom rumination, compulsive thinking, and reflective pondering exhibiting moderate correlations (0.644, 0.534, 0.491, all P<0.001). Rumination likely mediates the relationship between physical exercise and depressive symptoms, with the mediating effects of physical exercise differing across various rumination factors.

This study found that rumination can mediate the relationship between physical exercise and depressive symptoms. Among higher education students, physical exercise plays an important role in both physical and mental health (28), and students who exercise experience less academic stress compared to those who do not (29), and students who exercise experience less academic stress compared to those who do not (30). However, the effect of physical exercise on depressive symptoms is not direct; it requires mediation through rumination. Previous research has shown that lower habitual rumination and higher coping self-efficacy can partially mediate the impact of exercise habits on emotional symptoms and stress (31). Physical exercise directly influences the activity of the prefrontal cortex (32), which is a brain region associated with higher-order cognition, including rumination (33). This provides a physiological pathway for the effects of physical exercise on rumination. Bernstein and McNally demonstrated that even acute exercise performed before a stressor can buffer against emotion regulation dysfunction (including rumination), thereby mitigating negative emotional effects. They suggest that exercise reduces rumination symptoms, which would otherwise prolong negative emotional impacts (34, 35). Habitual ruminators may focus more on negative material and may reinforce negative memory biases. These individuals may require prolonged exercise programs to sustainably increase prefrontal downregulation of the limbic system. Rumination may serve as both a mechanism of exercise’s clinically relevant effects and as a moderating factor.

This study additionally identified that the intensity and frequency of physical exercise, along with the symptom rumination factor, serve as mediating pathways in this relationship. On one hand, the intensity and frequency of physical exercise may represent crucial factors influencing the mediating pathways. A randomized controlled trial revealed that college students exhibit a preference for high-intensity interval training (HIIT), potentially due to their inclination towards high-intensity yet shorter-duration exercise. Repeated sprints are particularly well-suited for young college students. This study effectively demonstrated the feasibility of HIIT in alleviating anxiety and depression among college students in the university context (36). A cross-sectional study published in *The Lancet* indicated a U-shaped relationship between exercise frequency and the mental health burden. Individuals engaging in exercise 3 to 5 times per week experienced a lower mental health burden compared to those exercising either less than 3 times or more than 5 times per week (37). Therefore, exercise at specific intensities and frequencies may serve as a more effective clinical target for mitigating mental health burdens. Conversely, symptom rumination may represent a significant mediating factor. Patients exhibiting depressive symptoms who engage in excessive rumination activate distributed neural circuits within the hippocampus and prefrontal cortex, thereby impairing their ability to form new associative memories. Physical exercise facilitates participants’ focus and attention, allowing them to acquire new cognitive skills, which subsequently reduces interference from negatively biased memories (23, 38). However, in this study, neither compulsive meditation nor reflective pondering demonstrated a correlation with physical exercise, potentially attributable to the exercise habits of college students. This observation warrants further investigation in future research. Notably, symptom rumination exhibited a significant correlation with depressive symptoms, providing robust evidence for the influence of rumination on depressive symptoms and offering a potential pathway for physical exercise to alleviate such symptoms in college students.

What we already know about this topic and what the current study adds to the existing body of knowledge. Previous research has shown that physical exercise is a significant negative predictor of depression among college students. However, related psychological factors appear to vary. Some studies indicate that student engagement in exercise and heightened sensitivity to behavioral activation strategies and reward information may play crucial roles in preventing and alleviating depressive symptoms (39). Additionally, studies have demonstrated that self-esteem and positive psychological capital serve as mediators, exerting a cascading influence on depressive symptoms in college students (40). Moreover, self-concept and social support also act as mediators in the relationship between physical exercise and depression among college students (41, 42). However, some studies suggest that the direct effect of physical exercise on depression in college students is not significant, though the indirect effect is substantial. Through independent mediating effects of mindfulness and meaning in life, physical exercise can significantly predict depressive symptoms in college students (43). These studies have all examined improvements in psychological factors.

We reviewed recent studies, and although Olson, Ryan L (22). proposed an aerobic exercise improvement plan in 2016 consisting of three 45-minute sessions per week, the exercise elements in this plan were not derived from rigorous experimental comparisons. In other words, only the effectiveness of aerobic exercise was assessed, which does not imply that all forms of exercise can be referenced. Alderman, B. L (23). also demonstrated the benefits of physical exercise. However, cognitive changes—such as the impact on different dimensions of rumination—and the exercise variables of type, frequency, duration, intensity, and time have yet to be further explored. The latest meta-analysis shows that, for alleviating depressive symptoms, exercise interventions lasting 30 to 60 minutes are the most effective. However, no clear conclusions have been made regarding exercise intensity (44). This raises further questions for us about which specific exercise elements contribute to improvements in depressive symptoms and which specific factors in rumination affect depressive symptoms.

Additionally, implementing a rigorous exercise regimen can be challenging for college students with depressive symptoms, as many students experience episodic depressive states. For most college students, managing exercise elements in their daily routine is more practical than following a strict exercise regimen. For example, college students who already engage in regular physical activity may find it easier to adjust exercise intensity or increase the duration of single sessions rather than adhere to a strict exercise schedule.

Limitations and future trends of the study: We have to admit that although we have controlled more influential factors and included as many samples as possible, there are still some problems that need to be paid attention to. At present, the diagnosis of the population is carried out by the self-rating scale of BDI-II. This may lack a clear diagnosis from a health care provider, so we can’t draw conclusions about depression. At the same time, due to the defects of the cross-sectional study design, we cannot draw the conclusion of causality, and the current view can only infer the hypothesis. In future studies, further cohort studies are needed to verify the views proposed in this study. Finally, in the future research direction, the model proposed in this study will be further verified through longitudinal studies to provide references for the formulation of clinical exercise programs, such as the formulation of exercise programs based on the intensity and duration of physical exercise.

## 5 Conclusion

In conclusion, rumination thinking and depression symptoms of college students are positively changed with physical exercise. Physical exercise can influence depression symptoms through the mediating effect of rumination thinking. The intensity, duration and symptom rumination of physical exercise are important ways to realize this, which provides a reference for the formulation of clinical exercise prescriptions.

## Data Availability

The raw data supporting the conclusions of this article will be made available by the authors, without undue reservation.
